# Application of denitrifying wood chip bioreactors for management of residential non-point sources of nitrogen

**DOI:** 10.1186/s13036-017-0057-4

**Published:** 2017-05-01

**Authors:** E. V. Lopez-Ponnada, T. J. Lynn, M. Peterson, S. J. Ergas, J. R. Mihelcic

**Affiliations:** 1 Department of Civil & Environmental Engineering, University of South Florida, 4202 E. Fowler Ave./ENB 118, Tampa, FL 33620 USA; 2 Texas A&M University-Kingsville, 700 University Blvd./MSC 213, Kingsville, TX 78363 USA

**Keywords:** Eutrophication, Green infrastructure, Low impact development (LID), On-site wastewater treatment, Best management practice (BMP), Biofilm, Septic systems, Stormwater, Decentralized treatment, Microbiology

## Abstract

Two important and large non-point sources of nitrogen in residential areas that adversely affect water quality are stormwater runoff and effluent from on-site treatment systems. These sources are challenging to control due to their variable flow rates and nitrogen concentrations. Denitrifying bioreactors that employ a lignocellulosic wood chip medium contained within a saturated (anoxic) zone are relatively new technology that can be implemented at the local level to manage residential non-point nitrogen sources. In these systems, wood chips serve as a microbial biofilm support and provide a constant source of organic substrate required for denitrification. Denitrifying wood chip bioreactors for stormwater management include biofilters and bioretention systems modified to include an internal water storage zone; for on-site wastewater, they include upflow packed bed reactors, permeable reactive barriers, and submerged wetlands. Laboratory studies have shown that these bioreactors can achieve nitrate removal efficiencies as high as 80–100% but could provide more fundamental insight into system design and performance. For example, the type and size of the wood chips, hydraulic loading rate, and dormant period between water applications affects the hydrolysis rate of the lignocellulosic substrate, which in turn affects the amount and bioavailability of dissolved organic carbon for denitrification. Additional field studies can provide a better understanding of the effect of varying environmental conditions such as ambient temperature, precipitation rates, household water use rates, and idle periods on nitrogen removal performance. Long-term studies are also essential for understanding operations and maintenance requirements and validating mathematical models that integrate the complex physical, chemical, and biological processes occurring in these systems. Better modeling tools could assist in optimizing denitrifying wood chip bioreactors to meet nutrient reduction goals in urban and suburban watersheds.

## Background

Discharge of excess nitrogen to coastal water bodies has led to increasing eutrophication and aquatic dead zones worldwide [1–3]. Managing the nitrogen cycle has been identified as a Grand Challenge by the U.S. National Academy of Engineering (NAE) [4] and imbalances in this cycle are recognized as having harmful effects on human health and the environment [2, 5]. Significant advances have been made in improving biological nitrogen removal (BNR) processes to manage point sources of nitrogen [6]. However, non-point sources of nitrogen from residential areas, such as stormwater runoff and discharges from on-site wastewater treatment systems, are difficult to control due to their diffuse nature and highly variable flow rates and concentrations. Over the last four decades, these non-point sources have also become a larger percent of the overall nitrogen loading to many coastal waters [1, 3, 7, 8]. For example, approximately 10 trillion gallons of untreated stormwater runoff end up in U.S. waterways, which are sources for water supply and recreation [9], and approximately 60 million people in the U.S. are currently served by on-site septic systems [10].

**Fig. 1:**
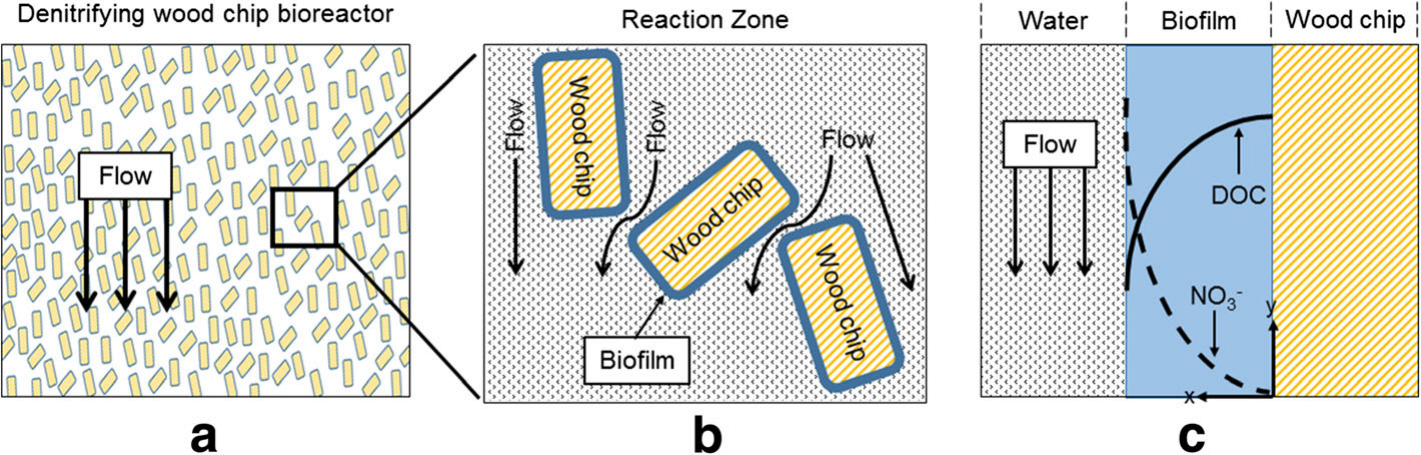
Denitrifying wood chip bioreactor schematics: **a** flow through the submerged (anoxic) zone, **b** biofilm on the wood chip support medium, **c** DOC dissolution and denitrification in the biofilm (x-axis:depth of biofilm and distance from wood chip, y- axis: concentration)

Denitrifying wood chip bioreactors are a viable management tool for control of non-point nitrogen sources in urban and suburban watersheds [1, 11, 12]. These bioreactors employ a submerged zone containing wood chips to promote denitrification [13, 14]. As shown in Fig. 1, the wood chips serve as both a microbial biofilm support and a source of dissolved organic carbon (DOC), which promotes a suitable environment for the growth of heterotrophic denitrifying bacteria [15]. The use of wood chips has been compared with other solid organic substrates for biological denitrification (e.g., maize cobs, wheat straw, green waste, sawdust) and have been found to be the most suitable for maintaining a steady NO_3_^−^ removal, limiting excessive DOC discharges and N_2_O emissions [16–19].

Denitrifying wood chip bioreactors are designed so stormwater or wastewater that enters the bioreactor encounters anoxic conditions that supports denitrification. An advantage of using a solid organic substrate in the bioreactor is that it eliminates the need to provide a liquid feed system for providing chemicals such as methanol, which can be an added expense and is difficult to handle, deliver, and store [6]. It is also an important and challenging task to supply the proper stoichiometric requirement of chemical inputs under dynamic loading conditions often observed in management of residential stormwater and on-site wastewater. Excessive input of organic substrate can result in carry-over of DOC to the effluent, while too little substrate can result in incomplete denitrification, both negatively affecting the environment [6]. Moreover, lignocellulosic materials are usually available at the local level, minimizing transportation costs. Other societal benefits associated with these nitrogen management technologies include reduced flooding, improved groundwater recharge, the potential for on-site reuse of treated water, incentives and credits to municipalities for increased nitrogen removal, and lower capital and operation and maintenance (O&M) costs [13, 20].

Denitrifying bioreactors that employ a lignocellulosic wood chip media are a promising technology for treatment of non-point sources of nitrogen in residential areas. However, identification of key knowledge gaps has not yet been performed that could lead to transformative advances of this technology. Accordingly, the objective of this paper is to provide a critical review of the literature on denitrifying bioreactors employing wood chip media used to manage residential non-point sources of nitrogen, specifically applications for stormwater runoff and on-site wastewater treatment. Prior review articles have focused on the use of denitrifying wood chip bioreactors for treatment of agricultural runoff [17, 21–24]. Those studies informed, but were not the focus of this review. Furthermore, although lignocellulosic wood chips are used in a number of other environmental applications, including bioremediation of acid mine drainage [25], biological air pollution control systems [26, 27], and treatment of aquaculture wastewaters [28], these topics are not discussed here.

## Applications of denitrifying wood chip bioreactors

As mentioned previously, in many residential areas, the two largest non-point sources of nitrogen are stormwater runoff and on-site wastewater [3, 29, 30]. Although the application and regulatory requirements for systems treating these sources are different, the denitrifying wood chip bioreactors used for both sources are similar in their design, operation and challenges. For example, both sources have highly variable influent flow rates, pollutant influent concentrations, and chemical forms of nitrogen (which includes ammonium [NH_4_^+^], nitrite [NO_2_^−^], nitrate [NO_3_^−^], dissolved organic N [DON] and particulate organic N [PON]). Because of seasonal variations in rainfall or household occupancy, these system experience long dormant periods, which can adversely impact microbial communities carrying out biological processes [31, 32]. This section thus describes denitrifying wood chip bioreactor configurations for managing these sources of nitrogen.

### Biofiltration systems for treatment of stormwater runoff

Sources of nitrogen in residential stormwater runoff include fertilizer from lawns, atmospheric deposition from stationary and mobile combustion sources, soil, pet waste, and other organic debris [33, 34]. Nitrogen concentrations and species in residential runoff vary because of regional and environmental factors such as climate, land use, housing density, and the distribution of air pollution nitrogen sources [35]. Typical total nitrogen (TN) concentrations in U.S. stormwater runoff are reported to be 2.0 mg N/L [36]. However, based on land use considerations, TN concentrations can range from 1.0 mg N/L for landscapes that maintain wetland and forest features to 2.4 mg N/L for landscapes that contain more impervious surfaces [37]. High-density residential areas also experience increases of TN in stormwater runoff to 11.6 mg N/L during the dry season when nutrients have had time to accumulate on impervious surfaces [38].

**Fig. 2:**
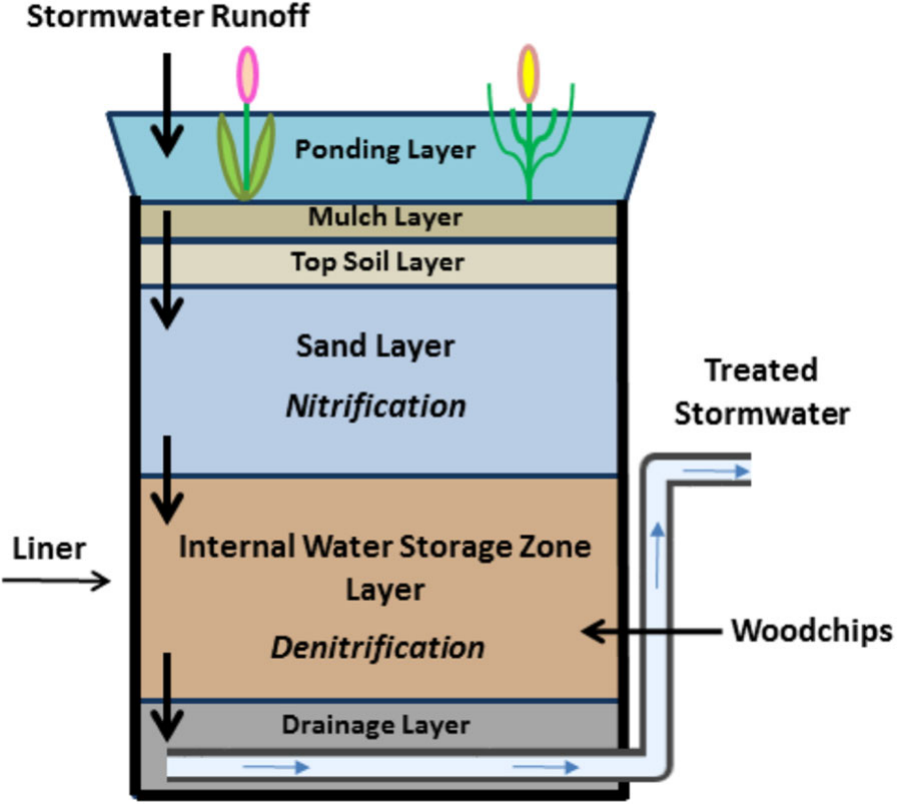
Six distinct zones in a modified bioretention unit. Top to bottom shows regions of stormwater ponding, mulch, top soil, nitrification, denitrification (IWSZ) and drainage layers. Wood chips are contained in the denitrification (IWSZ) zone

**Fig. 3:**
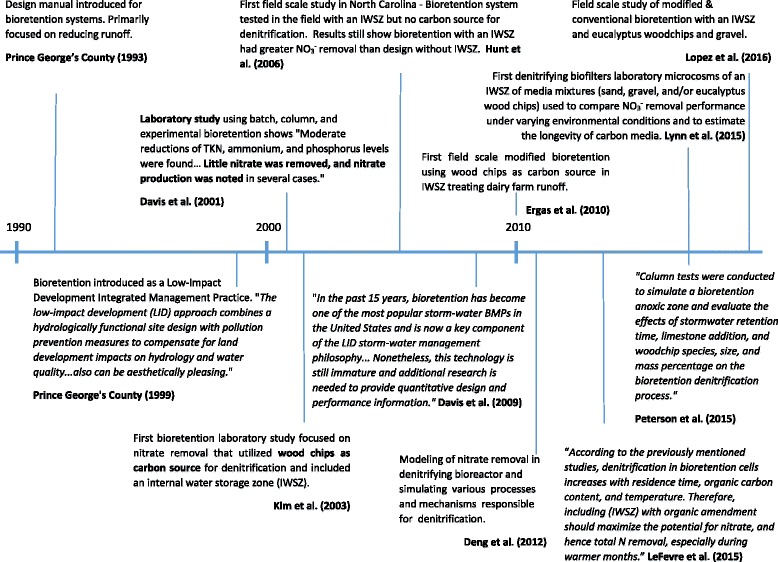
Timeline of design and research advances for bioretention systems

Biofilters, biofiltration systems, and bioretention systems (Fig. 2) are similar technologies whose names are used interchangeably in the literature. These are considered a low impact development (LID) technology and structural best management practice (BMP) used for stormwater management. Figure 3 provides a timeline of the design and research advances for bioretention systems and shows that the introduction of woodchips The first bioretention manual came out in 1993 in Maryland [39] (Fig. 3). LIDs attenuate peak flows and improve the quality of stormwater runoff before it enters receiving groundwater and/or surface water. LID technologies are designed to restore or preserve the natural hydrology of a site to before predevelopment conditions by working with the landscape, maintaining natural drainage courses, and reducing imperviousness [40, 41]. Structural LID technologies also include green roofs, permeable pavement, bioswales, and rainwater harvesting. Collins et al. [42] reviewed TN removal in eight types of stormwater control measures, including conventional and LID technologies (Table 1). In their study, they found that modified bioretention systems ranked highest for TN removal at 54.2%, while green roofs and permeable pavement ranked lowest, at 7.4 and −2.4% (TN export), respectively.

**Table 1:** Concentration-based TN removal efficiencies (%) for four low impact development (LID) technologies (adapted from Collins et al. [42])

LID Technology^a^	Median
Green roofs (*n* = 9)	7.4%
Permeable pavement (*n* = 5)	−2.4%
Bioretention - Conventional (*n* = 17)	25%
Bioretention - Modified (*n* = 5)	54.2%

^a^ *n* is the number of studies

Bioretention systems typically include plants (Fig. 2), which promote uptake of nutrients, enhance microbial activity in the root zones and contribute DOC for denitrification [42–44]. A timeline showing the development of bioretention systems is provided in Fig. 3. The application of wood chips into an internal water storage zone (IWSZ) is a relatively new feature of these systems. Bioretention systems are relatively shallow depressions with a planting bed where stormwater runoff slowly infiltrates through different permeable layers such as vegetated soil, sand and gravel (Fig. 2). Conventional bioretention systems have been shown to achieve high removal efficiencies for suspended solids, organics, metals, and phosphorus through sedimentation, filtration, adsorption and plant and microbial uptake [23, 35]. However, a number of studies have shown poor TN removal, with an average of 25% (Table 1), and at times the effluent TN concentrations have been reported to exceed the influent concentrations [1, 42]. This is because conventional bioretention systems typically operate under unsaturated down flow hydraulic conditions, which promotes an aerobic environment. Under these conditions, NH_4_^+^ is oxidized to NO_2_^−^ and NO_3_^−^ via nitrification and exported with the effluent [1]. Dissolved organic nitrogen that leaches from the system may also originate from mulch, compost, soil or decaying plant matter [35, 45].

Although, nitrogen removal efficiencies for conventional bioretention systems studied at the laboratory and pilot-scale are reported to range from 50–75% for total Kjeldahl nitrogen (TKN) and 60–80% for NH_4_^+^ [33], the export of NO_2_^−^ + NO_3_^−^ (NO_x_) and DON often negates the more effective removal of PON and NH_4_^+^ [33]. For example, a net export of 630% of NO_x_-N [46] was reported for a conventional bioretention system with organic material placed in the top layers and another laboratory study reported a net export of 204% NO_3_^—^N [47] for a conventional bioretention with plants and shredded hardwood bark as mulch. The National Pollutant Removal Performance Database reports median NO_x_ and TN removal efficiencies for field studies of conventional bioretention systems of only 43 and 46%, respectively [48].

The highly variable TN removal efficiencies observed in conventional bioretention systems led to the development of modified biofilters or modified bioretention systems (previously shown in Fig. 2). Kim et al. [16] first proposed a modification of a conventional biofiltration system to include an internal water storage zone (IWSZ) containing an electron donor such as wood chips to facilitate denitrification. In these systems, an upturned elbow maintains saturated conditions in the wood chip zone (Fig. 2), which limits oxygen diffusion to the biofilm, creating the anoxic conditions required for denitrification. Kim et al. [16] and Hunt [49] reported that TN removals > 80% could be achieved in laboratory columns with such system. The first field study of a modified bioretention system was in Connecticut and showed TN removal efficiencies of approximately 82% when these systems treated dairy farm runoff.

The long retention time in the IWSZ during antecedent dry conditions (ADCs, the number of dry days between storm events [50]) facilitates dissolution of DOC from the wood chips and denitrification during the dormant period [32]. Laboratory column experiments of an IWSZ filled with eucalyptus wood chips and gravel (1:2 by volume) demonstrated > 80% removal of TN [32]. With the same medium, ~100% removal of NO_3_^−^ was achieved in acclimated anoxic microcosms within 6 h. The design of a modified bioretention system and selection of the media are thus both important for the efficiency of TN removal.

Reviews of the performance of bioretention systems in the field [1, 51] identified 15 conventional and 7 modified bioretention systems (Table 2). Field studies have been conducted in only six (primarily eastern) U.S. states (Maryland, Connecticut, North Carolina, Virginia, Florida, and Washington) and Australia, with modified bioretention system field studies limited to four eastern states. Although research on modified bioretention systems has been on-going since 2003 (Fig. 3), only two studies have used wood chips at the field scale ([23, 52]: Table 2). Additional field studies are necessary to provide design guidance for implementing modified bioretention systems in different climate zones (e.g., arid, sub-tropical and tropical), with different seasonal sunlight and precipitation patterns, and with different native and common ornamental plants and locally available lignocellulosic materials.

**Table 2:** Field studies focused on removal of dissolved nutrients from stormwater and agricultural runoff with a modified bioretention system (adapted from LeFevre et al. [1])

Study #	Location	Carbon Source for Modified System	Lined	U.S. Climate Regions defined by NOAA	Reference
1	Maryland	Shredded newspaper	Yes	Northeast	[102]
2	North Carolina	Not specified. Assuming organic material in fill soil media	Yes	Southeast	[103]
3	Maryland	Shredded newspaper	Not specified	Northeast	[104]
4	North Carolina	Not specified. Assuming organic material in fill soil media	No	Southeast	[105]
5	North Carolina	Assuming organic material in fill soil media	No	Southeast	[106]
6	Connecticut	Wood chips (maple and birch wood)	Yes	Northeast	[23]
7	Florida	Wood chips (eucalyptus wood)	Yes	Southeast	[52]

### Denitrifying wood chip bioreactors for on-site wastewater treatment

On-site wastewater is also referred to as domestic wastewater, residential wastewater, domestic sewage, or a combination of these terms. For simplicity, the term residential wastewater is used here and refers to all the wastewater collected from a residence including water from toilets, showers, kitchen sinks and laundry. Conventional on-site residential wastewater treatment systems consist of a septic tank for solids separation followed by a soil infiltration system (or drain field), which provides further biological treatment and some pathogen removal. Advantages of on-site wastewater treatment include their simplicity of operation, low installation cost, low O&M requirements and the ability to recharge local groundwater resources [53]. Major challenges of on-site wastewater treatment systems include siting restrictions in areas with a high groundwater table and proximity to drinking water sources and environmentally sensitive areas [54]. As discussed previously, on-site wastewater treatment systems are also subject to highly variable hydraulic and pollutant loading rates and long idle times (e.g., during vacations or seasonal use). In addition, these systems mainly depend on the homeowner to carry out or schedule required maintenance.

Most of the nitrogen in residential wastewater is in the form of PON, DON and NH_4_^+^. Although nitrification is observed in aerobic regions of the drain field, conditions in conventional on-site wastewater treatment systems do not favor denitrification, resulting in NO_3_^−^ contamination of surface water and groundwater. Mechanical systems that require outside input of electricity that are similar to a centralized activated sludge BNR processes have been developed to improve nitrogen removal in on-site wastewater treatment systems. However, studies of these mechanical systems have shown inconsistent performance, with generally less than 60% TN removal, and problems due to the lack of O&M requirements by homeowners [55–57].

**Fig. 4:**
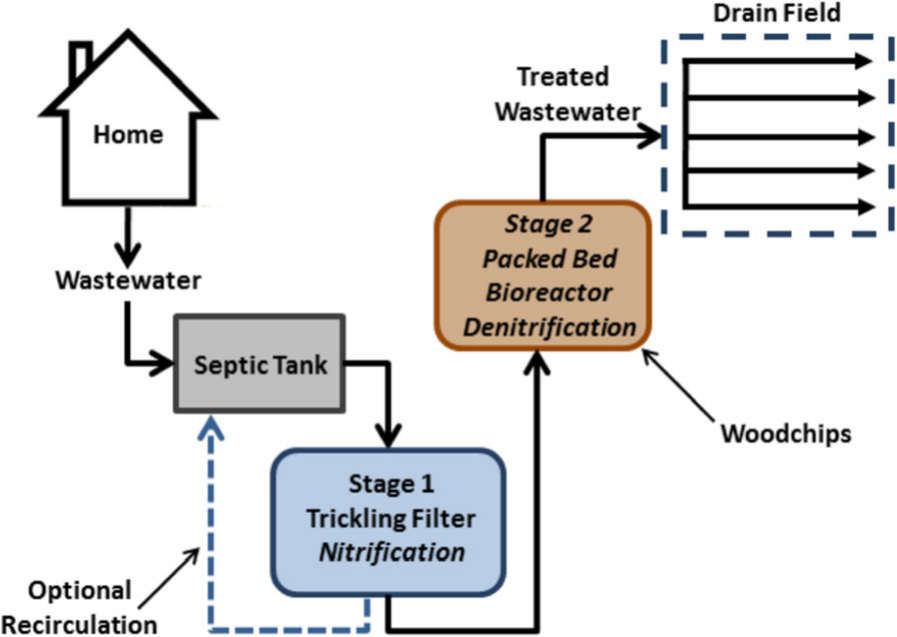
Schematic of a residential on-site wastewater treatment system employing a denitrifying wood chip bioreactor (Stage 2)

Because of these challenges, “passive” BNR systems have been developed for on-site wastewater treatment that are similar to conventional septic systems in their O&M requirements [58]. Figure 4 shows an example of a passive on-site wastewater BNR system. Nitrification takes place in the first stage, which consists of an unsaturated trickling filter containing sand, expanded clay, gravel or zeolite media [59]. Denitrification takes place in a second stage, which consists of a packed bed reactor containing a “reactive” medium, such as wood chips [60] or elemental sulfur pellets [61]. Recirculation of effluent from the trickling filter back to the septic tank or a separate pre-anoxic tank is often used to dilute the influent to Stage 1 and reduce the influent NO_3_^−^ loading to Stage 2 [62]. Similar to modified bioretention systems that manage stormwater, submerged conditions are normally maintained in the wood chip reactor to favor the development of anoxic conditions while the reactive medium serves as both a microbial biofilm carrier and organic carbon substrate for denitrification (Fig. 1). Several of these systems are available commercially, including Nitrex™ (supplied by Lombardo and Associates, Newton, MA) and De-Nyte® (supplied by Presby Environmental, Whitefield, NH) technologies. Other wood chip based denitrification technologies for on-site wastewater treatment include systems that combine nitrification and denitrification stages within a single unit [63], permeable reactive barriers (also called denitrification walls [64]) and horizontal or vertical flow wetlands containing wood chips [65, 66].

**Fig. 5:**
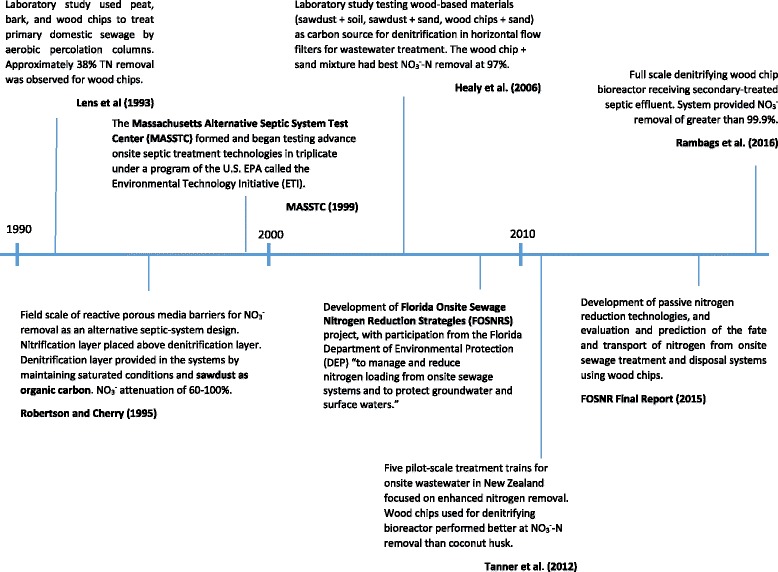
Timeline of research and design advances for on-site wastewater treatment

A timeline showing the development of wood chip bioreactors for on-site wastewater treatment is provided in Fig. 5. This timeline suggests the field performance of a wood chip bioreactor for on-site wastewater management is more advanced when compared to stormwater management. Lens et al. [31] carried out studies of treatment of unsettled wastewater in bench scale columns containing peat, bark and wood chip media. Approximately 38% TN removal was observed with wood chips even though the systems were operated as aerobic percolation columns and were not specifically designed for denitrification. Another bench-scale study evaluated pine sawdust, sawdust mixed with soil, and wood chips/sand media for wastewater denitrification in horizontal-flow filters with 26 day empty bed contact times (EBCT = reactor volume/flow rate) [60]. In that study, the wood chip and sand mixture (1:1 ratio by volume) yielded the best NO_3_^−^ removal performance (>97%). However, daily addition of sodium sulfite (a dissolved oxygen [DO] scavenger) was required to maintain anoxic conditions in the column. Nitrified residential wastewater was treated in a packed bed reactor containing a mixture of wood chips and sawdust [13], resulting in consistently low effluent NO_3_^−^ concentrations with little export of organic carbon. Tanner et al. [67] investigated five different treatment trains for on-site wastewater treatment and concluded that the best overall TN removal (95%) was obtained when a recirculating vertical flow wetland with a sand medium was followed by a packed bed reactor containing wood chips. Rambags et al. [68] sampled a full-scale wood chip denitrifying bioreactor receiving secondary-treated septic tank effluent. Greater than 99.9% removal of NO_3_^−^ was observed, along with high removal efficiencies for total suspended solids (TSS), fecal indicator bacteria and viruses; however, removal of NH_4_^+^, organic nitrogen, and phosphorus was inconsistent.

Several studies have investigated the use of permeable reactive barriers for on-site wastewater denitrification. In these systems, a permeable wall of wood chips is constructed in the subsurface downstream of the drain field to intercept the NO_3_^−^ contaminated groundwater plume. One study observed almost complete denitrification using this approach [64]. Additional studies have been carried out using horizontal flow, vertical flow, and hybrid wetlands systems containing wood chip media that might be useful to guide research for on-site wastewater management. For example, a hybrid wetland system was tested that consisted of a vertical flow wetland with wood chips, followed by a horizontal flow wetland with gravel and finally a vertical flow wetland with zeolite [65]. The observed removal of TN (72%) was attributed to both high oxygen transfer for nitrification and organic carbon availability from the wood chips for denitrification. The same authors also compared hybrid systems consisting of vertical flow followed by horizontal flow wetlands with different types of media (gravel, wood chip and a gravel wood chip mixture) [69]. Improved TN removal performance was observed in the systems containing wood chips compared to the traditional system with only gravel, with 98% TN removal in the vertical flow system [69].

## Process microbiology

Nitrogen transformation processes that occur in denitrifying wood chip bioreactors include uptake of nitrogen by plants (assimilation) and microorganisms, nitrification, denitrification, dissimilatory reduction of nitrate to ammonia and anaerobic ammonia oxidation (ANAMMOX). Several studies have reported that dissimilatory reduction of nitrate to ammonia plays only a small role in NO_3_^−^ removal in denitrifying wood chip bioreactors [18, 22, 70], while little research has been carried out on the role of ANAMMOX in these systems, therefore these processes are not discussed further. Because the electron donor for denitrification is primarily obtained from the wood chips, factors affecting the hydrolysis of lignocellulosic biomass are discussed here.

### Nitrogen transformation processes

Nitrogen is an important macronutrient that is taken up from the soil and incorporated into plant and microbial biomass. Several studies have compared bioretention systems with and without plants and found that generally, systems with plants perform better at removing nitrogen than systems without plants [42, 44, 71, 72]. Studies have observed how plant species along with the organic content in the media of the bioreactor and the use of an IWSZ influence the variation of nitrogen removal. In some instances nitrogen leaching has occurred, attributed mainly to leaching of nitrogen from organic matter in the soil [71]. In addition, the presence of plants enhances microbial activity in the root zones, more aerobic conditions for nitrification and contributes DOC for denitrification [71, 73].

Both modified bioretention systems (Fig. 2) and denitrifying wood chip bioreactors for on-site wastewater treatment (Fig. 4) are often designed to include an unsaturated zone for nitrification prior to denitrification. Nitrification is an aerobic process, requiring sufficient DO for oxidation of NH_4_^+^ to NO_2_^−^ by ammonia oxidizing bacteria and archaea, followed by oxidation of NO_2_^−^ to NO_3_^−^ by nitrite oxidizing bacteria. Nitrification performance can be limited by low contact times at high hydraulic loading rates, washout of microorganisms (e.g., due to high shear forces), low temperatures, in low alkalinity waters, insufficient oxygen transfer to the nitrifying biofilm, and due to the presence of toxic organic compounds and metals [6]. For example, in a laboratory and field study of modified bioretention systems, nitrification appeared to limit TN removal, since TN and NH_4_^+^ concentrations were high yet NO_3_^−^-N concentrations were below detection limits, indicating complete denitrification [23].

In denitrification, facultative microorganisms respire NO_3_^−^ or NO_2_^−^ under anoxic conditions [74]; therefore saturated conditions that limit oxygen transfer and promote the development of an anoxic zone are normally included in denitrifying wood chip bioreactors. A variety of electron donors can be used for denitrification including inorganic compounds, such as elemental sulfur [61] and dissolved organic carbon leached from the wood chips as shown in Fig. 1 [32, 75]. Denitrification normally proceeds through a series of four sequential steps (NO_3_^−^ → NO_2_^−^ → NO(g) → N_2_O(g) → N_2_(g)). A number of genera of denitrifying microorganisms, as well as some archaea and fungi, have been identified including *Firmicutes*, *Actinomycetes*, *Bacteriodes*, *Aquifaceae*, *Proteobacteria Alphaproteobacteria*, *Betaproteobacteria*, *Gammaproteobacteria* and *Epsilonproteobacteria* [76].

Production of N_2_O is a particular concern for BNR processes because it is a potent greenhouse gas and ozone-depleting compound. Studies of denitrifying wood chip bioreactors have shown that N_2_O emissions are lower or similar to N_2_O emissions from fertilized agricultural fields or systems using other organic carbon sources [77]. For example, only a small fraction of the NO_3_^−^ removed (0.6%) from a full scale denitrifying wood chip bioreactor in Canada was emitted as N_2_O. N_2_O emission rates were comparable to those reported for agricultural croplands and less than emissions from nitrogen polluted water bodies [77]. In the summer months, the denitrifying bioreactor acted as an N_2_O sink [77]. Grover et al. [78] reported that bioretention systems were only minor N_2_O sources. Although peak N_2_O emissions from a modified bioretention system were an order of magnitude greater than from a conventional system, concentrations were the same magnitude as fertilized irrigated lawns [78]. Additional research is needed on characterizing N_2_O emissions from denitrifying wood chip bioreactors used to treat on-site wastewater, specifically studies that provide greater insights into the mechanisms of N_2_O production under transient loading conditions, and denitrifying wood chip bioreactor designs that minimize N_2_O emissions. Methane (CH_​4_) ﻿emissions may also be a concern because it is a potent greenhouse gas. ﻿Although CH_4_ emissions for denitrifying wood chip bioreactors are reported as lower than for constructed wetlands, conventional wastewater treatment, and manure composting facilities [77].

Several studies have investigated the presence of nitrogen transforming genes involved in denitrification in wood chip bioreactors [76, 79, 80]. Chen et al. [68] quantified nitrifying and denitrifying genes in the sand (nitrifying) and mulch layers of a conventional bioretention system. The results showed that the quantity of nitrifying and denitrifying genes decreased as a function of media depth, possibly due to decreases in DOC availability with depth [68]. In denitrification beds treating agricultural runoff it was concluded that microbial denitrification was the primary mechanism for NO_3_^−^ removal due to the abundance of cytochrome nitrite reductase (*nirS)* or copper nitrite reductase *(nirK)* genes [18].

### Biodegradation of lignocellulosic material

A general stoichiometric equation for denitrification using a simple carbohydrate (CH_2_O) as an electron donor can be written as:

1 $$ 5 C{H}_2 O+4 N{O}_3^{-}+4{H}^{+}\to 2{N}_2+5 C{O}_2+7{H}_2 O $$

The simple carbohydrate could be derived from natural organic solid substrates that include wood, compost, leaves, or soil organic matter [70]. Wood is primarily composed of lignocellulose that consists of cellulose (45–55% content), hemi-cellulose (24–40%) and lignin (18–35%) [81, 82]. Cellulose is a glucose polymer with α-1,4-linkages, hemicellulose is a heteropolysaccharide polymer and lignin is an amorphous heteropolymer [82, 83]. Use of a solid substrate requires the additional step of hydrolysis to first solubilize the organic carbon [15]. Hydrolysis occurs when bacteria excrete extracellular enzymes that break down solid substrates into DOC that has a small enough molecular weight to pass (or dissolve) through the bacteria’s cell membrane [84]. The rate of hydrolysis of hemicellulose is known to occur fastest, followed by cellulose and then lignin [83].

The biodegradation of cellulose, hemi-cellulose and lignin requires different enzymes and bacteria [84]. Cellulose is the most studied compound in mesophilic anaerobic environments, which is an expected operating environment for residential denitrifying bioreactors. Enzymes that depolymerize cellulose in these environments are organized in multi-enzymatic complexes called cellulosomes [85]. Enzymes found in cellulosomes are known to include endoglucanase, cellobiohydrolase and xylanase [86]. The products of cellulose depolymerization include cellobiose, cellodextrines and glucose, which can be metabolized in biofilms [85, 86].

Bacteria and fungi that are known to produce cellulosic hydrolytic extracellular enzymes have also been shown to exhibit other interesting capabilities that may be of importance in wood chip bioreactors [86]. *Clostriduium cellulovorans* is capable of utilizing other carbon sources found in wood, such as xylan (hemicellulose) and pectin [87]; the cellulosomes of *Clostridium cellulolyticum* are known to facilitate bacterial adhesion onto solid substrates [85]; in nitrogen-limited environments, *Cellulomonas spp.* can utilize NH_4_^+^ from solid cellulosic substrates for synthesis [88].

### Effect of transient loading conditions on microbial processes

Differences have been observed in effluent water quality during start-up, operation, and dormant phases of denitrifying wood chip bioreactors. This may be due to the growth of microbial biofilms on the wood chips as the bioreactors mature with time or changes in the availability of different terminal electron acceptors [31]. During the start-up phase, denitrifying wood chip bioreactors have been reported in some instances to export high concentrations of DOC and TKN and remove only a small amount of NO_3_^−^ [32]. This may be due to the presence of aerobic conditions initially in the bioreactor. Higher rates of hydrolysis of lignocellulosic material are observed under aerobic compared with anaerobic conditions [83, 86, 89], resulting in more leaching of DOC and DON from the system [32, 90]. In addition, performance is expected to improve as denitrifying biofilms are established in the reactors. The duration of the start-up phase for denitrification has been shown to be between six hours and one month [32]; however, the precise time-scale for start-up is unknown. Extended start-up periods are reported to be required for bioretention systems [23] and wastewater treatment systems [31] that included unsaturated zones for nitrification. Nitrifiers are slow growing autotrophs that require longer acclimation periods [6].

NO_3_^−^ removal rates increase as anoxic conditions are established, which facilitate the activity of denitrifying organisms [91]. In systems where both DO and NO_3_^−^ are present in the influent [32, 92] and a carbon source is available in excess, microbial communities will first utilize DO as an electron acceptor because it is more energetically favorable and then switch to NO_3_^−^ after DO is depleted below a certain level [74]. Lynn et al. [32] estimated an oxygen inhibition coefficient value for the Andrew’s equation of 2.2 mg/L in a wood chip stormwater biofilter microcosm study.

During operation, excess DOC washes out of the bioreactor pore water as the influent water “mixes” with the water retained in the bioreactor pore water [32]. This decrease in pore water DOC results in decreased NO_3_^−^ removal at high flow rates or longer periods of continuous operation [32]. At lower hydraulic loading rates, NO_3_^−^ removal rates increase as denitrifiers have more contact time to utilize NO_3_^−^ in the water.

During the dormant phase when the reactor is not receiving influent, NO_3_^−^ will become depleted, DOC concentrations will increase, oxidation reduction potential will decrease, and sulfate reduction can occur [24, 32, 77], resulting in odorous hydrogen sulfide production. Decreases in pore water DOC concentrations were observed after an extended dormant period (e.g., > 16 days) [32] possibly due to the growth of methanogens [77].

## Physical characteristics and operating conditions that impact design & performance

A number of factors influence the performance of denitrifying wood chip bioreactors including: (1) physical characteristics such as wood chip type and size and bioreactor depth; and (2) operating conditions such as hydraulic loading rate, hydraulic retention time (HRT), length of antecedent dry conditions, influent nitrogen concentration, temperature, other additives present in the media, media saturation and media longevity [13, 17, 32, 93].

### Wood chip type and size

The wood chip medium used in denitrifying wood chip bioreactors has been obtained from hardwood and softwood trees (Table 3). Hardwood trees have broader leaves and a higher carbon content and density than softwoods [94]. In general, observed TN removal rates are higher with softwood compared with hardwoods (Table 3). However, Peterson et al. [91] observed higher TN removals with the hardwoods Willow Oak and Red Maple than Virginia Pine softwood. These studies suggest that future research could determine the exact mechanism(s) that cause a particular wood chip type to influence the denitrification rate or long-term NO_3_^−^ removal performance. In addition, life cycle and economic assessments can assist our understanding of the environmental sustainability and cost of different materials.

**Table 3:** Collected data for nine different types of wood chips: type of study performed, carbon content, TOC leaching, influent and effluent nitrogen concentrations, and nitrogen removal

	Wood Type	Type of Study	Carbon Content (%)	Leached TOC (mg TOC/L)	Influent Concentration (mg N/L)	Effluent Concentration (mg N/L)	N – Removal (%)	Reference
Softwood	Pine	Column	47	100	3	1.56	48	[91]^a^
	Pine	Column	–	–	15.8	11.1	30	[18]
	Pine	Column	28	158	50	<2.0	96	[70]
	Pine	Column	28	175.3	50	17.7	65	[70]
	Pine	Column	50	–	26	1.8	93	[19]
	Pine	Batch	47	–	57.8	6.4	89	[75]
	Coniferous	Batch	44	–	32.2	1.6	95	[70]
	Willow	Batch	47	120	32.2	4.5	86	[70]
	Average		41.5 (9.3)	138.3 (34.4)	33.4 (18.7)	5.84 (5.8)	75.2 (24.9)	
Hardwood	Eucalyptus	Column	51	–	2.3	BDL	100	[32, 107] ^a^
	Eucalyptus	Column	–	–	15.8	9.9	37	[18]
	Maple	Column	49	42	3	1.1	62	[91]
	Maple/Birch	Pilot	–	–	7.6	0.9	88	[23]
	Red Gum	Batch	44	–	55	7	87	[75]
	Wild Cherry	Column	50	153	3	1.9	36	[91]
	Oak	Column	50	41	3	1.2	62	[91]
	Beech	Column	50	45	3	2	32	[91]
	Average		48.8 (2.5)	70.3 (55.2)	11.6 (18.1)	3.45 (3.6)	63.0 (26.6)	

Standard deviation (if applicable) is in parenthesis. *BDL:* below detection limit

^a^ Study that reported ADC

Two studies evaluated the effect of wood chip size on the performance of denitrifying bioreactors [17, 91]. Cameron and Schipper [17] reported a slight increase in NO_3_^−^ removal efficiency with increasing wood chip size but the difference was statistically insignificant. Larger sized wood chips may contribute to higher porosity in the bioreactor greater internal pore structure that may lead to greater water holding capacity of a reactor. In contrast, Peterson et al. [91] found that NO_3_^−^ removal efficiencies were higher with smaller wood chip sizes. Smaller wood chips have a higher total surface area per unit mass, leading to more area for biofilms to grow (Fig. 1). However, smaller wood chips would be expected to also leach more TKN, which can offset some of the improvements in overall nitrogen removal [91]. The results from these studies demonstrate how wood chip size influences a number of other factors (e.g., porosity of the IWSZ, DOC leaching rates) that can play a role in increasing or reducing NO_3_^−^ removal rates. In addition, the contradicting results for nitrogen removal with wood chip size may be due to the higher influent NO_3_^−^ concentration used in the wastewater [17] compared to the stormwater study [91].

### Saturated zone depth

The depth of the saturated zone can influence the performance of denitrifying biofilters for both stormwater and on-site wastewater treatment. Lynn et al. [7] studied denitrifying wood chip biofilters with varying depths that were operated with the same HRT. Greater NO_3_^−^ removal was reported in taller columns (45 and 60 cm) compared to a shorter column (30 cm) at HRTs ≥ 3 h. Tracer studies revealed that dispersion dominated transport was more pronounced in the shorter column. Similarly, a minimum IWSZ depth of 45 cm was reported by Zinger et al. [95] as optimal for TN removal. This same depth is included in the design depth recommendations by the Facility for Advancing Water Biofiltration [96]. This could potentially limit subsurface applications of denitrifying wood chip bioreactors in regions with high water tables or require larger overall volumes for shallower reactors. Thus, greater understanding is needed of the interplay between IWSZ depth, denitrification performance, and associated costs.

### Hydraulic loading rate

In a similar way that an increase in column depth improves NO_3_^−^ removal due to longer HRT, a decrease in hydraulic loading rate can also increase retention time and improve NO_3_^−^ removal. Hydraulic loading rates for stormwater and on-site wastewater are naturally variable, but they can be reduced when incorporating flow control devices at the bioreactor outlet [1, 97, 98]. Lucas and Greenway [97] installed a regulated outlet in bioretention mesocosms, which increased the HRT from 15 min as free flow discharge to about 150 min when regulated. The authors observed up to 2.7 times greater NO_x_ removal with increased retention time. Similarly for denitrifying bioreactors in the field, a regulated outlet control device could provide additional retention time for denitrification but additional ponding area storage capacity may be required if the influent flow rate is greater than the regulated effluent flow rate. For on-site wastewater, flow equalization or a decrease in water use within the household through more water efficient technologies or behavioral change could improve NO_3_^−^ removal.

### Intermittent conditions

Intermittent loading conditions in denitrifying wood chip bioreactors are due to variations in nitrogen concentrations and diurnal fluctuations in residential water use and/or varying precipitation patterns associated with stormwater runoff. Intermittent operation constantly changes the biochemical processes that influence nitrogen transformation and DOC dissolution (Effect of transient loading conditions on microbial processes section). The impact of intermittent operational conditions in these systems in not well studied and should consider differences in physical, chemical and microbial processes that influence nitrogen removal performance during start-up, operation, and dormant phases.

### Longevity

For practical application, the longevity of municipal denitrifying bioreactors is expected to be decades. Field studies performed on on-site wastewater treatment systems have reported appreciable denitrification activity after 15 years of operation [24, 99] and a microcosm study performed on stormwater denitrifying bioreactors estimated wood chip longevity of 21 years [7]. These findings fall within the estimated range of 9 to 72 years proposed for agricultural denitrifying bioreactors [14]. However, bioreactor saturation conditions may significantly affect bioreactor longevity. For example, a field study on agricultural denitrifying bioreactors observed increased wood chip degradation in an unsaturated-prone zone of a denitrification wall compared to a saturated-prone zone [21]. These results indicate that saturated conditions should be maintained to sustain the longevity of denitrifying wood chip bioreactors.

## Modeling of denitrifying wood chip bioreactors

Although several studies highlight TN removal in denitrifying wood chip bioreactors used for stormwater runoff or on-site wastewater, few studies have developed quantitative models to assess the overall TN reduction effectiveness or guide future research. Without these models, TN load reduction design standards may be unreliable and the flexibility of the designer may be limited to dimensionally “fit” these systems into unique site characteristics. For example, during large storm events, much of the untreated stormwater runoff may by-pass the denitrifying bioreactor by overflowing from the ponding area. This large volume of untreated runoff may result in low overall TN reductions for the system regardless of TN removal efficiency of the bioreactor. Likewise for on-site wastewater treatment systems, if not sized properly for the incoming flow and volumes, the intended efficiency of nitrogen removal may not occur. When developed, these models could be applied to other wood chip denitrifying bioreactors such as permeable reactive barriers or biofilters used to remove NO_3_^−^ from agricultural runoff. Two challenges in developing these models is the accurate modeling of complex nitrogen transformation processes that occur at the biofilm-scale and integrating these models into watershed-scale hydrological modeling programs for groundwater transport (on-site wastewater) or surface water transport (stormwater).

Current models for stormwater management, such as the U.S. Environmental Protection Agency (EPA) Stormwater Management Model (SWMM), RECARGA, and DRAINMOD, focus more on the hydraulics and hydrology of the system rather than water quality. Two studies have however developed models that address water quality for denitrifying stormwater bioreactors. Deng et al. [100] developed a model for bioreactors containing different organic carbon amendments and included processes for dispersion, mass transfer of NO_3_^−^ into the biofilm, microbial growth, oxygen inhibition, DOC substrate limitation and temperature. This model may be useful for investigating NO_3_^−^ rate limiting factors that occur within microbial biofilms. A denitrification model that is compatible with SWMM version 5.1 has also been developed that can be used when designing stormwater management systems for land development projects [50]. The processes included in that model are wood chip dissolution and a denitrification kinetic model that incorporates DO and bioavailable DOC. This model may be useful for simultaneously evaluating water quality (e.g., NO_3_^−^ removal) and water quantity (e.g., runoff volume/rate reduction) goals based on a stormwater system design. Although the model predicted NO_3_^−^ removal within 10% of experimental results and is validated with a high Nash-Sutcliffe efficiency coefficient of 0.8, it was recommended that the model be validated and calibrated with field data. These two denitrification models [50, 100] could also be improved by integrating a nitrification component to quantify TN load reduction effectiveness according to the specified use (e.g., on-site or stormwater) and bioreactor geometries. Advancement of knowledge on biological process within the different layers of denitrifying wood chip bioreactors can also improve modeling efforts to assist in watershed scale studies and the impact of implementing these systems at hotspots for nitrogen or sensitive ecosystems [11].

## Conclusions

Denitrifying wood chip bioreactors can assist in removing nitrogen from non-point sources of residential pollution, such as stormwater runoff and on-site wastewater. The wood chip medium (a lignocellulosic substrate) provides a support structure for biofilms and the organic carbon source required for heterotrophic denitrifying bacteria that is essential for the transformation of reactive nitrogen to unreactive dinitrogen gas. Advantages of these passive systems are that they can handle the highly variable flow rates and nitrogen concentrations observed in stormwater runoff and on-site wastewater treatment. The use of a solid organic substrate obviates the need for liquid chemical feed systems and reduces the risk of carry-over of excess organic carbon into the effluent. Denitrifying wood chip bioreactors are considered appropriate technologies because they have minimal mechanical energy and chemical inputs and use plant-based and locally available materials such as wood chips, sand, and gravel. In addition, they provide benefits of groundwater recharge and opportunities for water reuse close to the site of wastewater generation.

Biofilters and bioretention systems that include an IWSZ containing wood chips achieve improved nitrogen removal from stormwater runoff than conventional BMPs. The performance of these systems depends largely on hydraulic and pollutant loading, which fluctuate with individual storm events and seasonal use and precipitation and thus is dependent on geographic location. However, little research has examined the performance of these systems under dynamic loading conditions in different climates, such as arid or tropical climates. With increasing changes in climate and more extreme weather events influencing precipitation and antecedent dry conditions, additional field studies that are linked to modeling will help understand the long-term performance and potential benefits of these systems [101].

A number of different denitrifying wood chip bioreactor process configurations have been successfully used to remove nitrogen from on-site wastewater, including packed bed reactors, permeable reactive barriers and submerged wetlands, with and without recirculation. Continuing long term studies on the dynamic performance of these systems would provide consistent and long-term nitrogen removal efficiency. Also, use of life cycle assessment and life cost analysis could assist efforts to quantify the economic and environmental tradeoffs between on-site nutrient removal versus expansion of sewers and centralized wastewater treatment systems for rural and suburban areas.

The type and size of the wood chips, hydraulic loading rate, and dormant period between periods of water application (e.g., during storm events or residential water use) have been shown to affect the hydrolysis rate of the lignocellulosic substrate, which affects the amount and bioavailability of DOC for denitrification. Maintaining saturated conditions during non-operational periods is also a critical design feature that controls the overall performance of denitrifying bioreactors. Higher NO_3_^−^ removal, lower TKN export and longer wood chip media longevity is expected from these designs compared with bioreactors that are only designed for saturation during operation. Future research could focus on understanding the interrelationships between bioreactor parameters and developing mathematical models and design tools that can be used to quantify water quality and quantity performance as a function of varying bioreactor designs and environmental conditions. Most studies of wood chip bioreactors have been performed on the individual performance of bench- or field-scale units rather than evaluating the impact of multiple systems on ground and surface water quantity and quality within a watershed. Lastly, incorporating life cycle assessments and life cost analysis studies on multiple systems can provide a holistic overview of the sustainability of implementing these systems at the watershed scale.
